# High-risk behaviors among commercial drivers: a cross-sectional study in Peshawar, Khyber Pakhtunkhwa

**Published:** 2019-08

**Authors:** Irfan khattak

**Affiliations:** ^*a*^Epidemiology & Biostatistics, Khyber Medical University, Pakistan.

## Abstract

**Background:**

Road traffic accidents (RTAs) are the leading cause of death in the world in the young age category. Pakistan is a populous country with a huge number of vehicles on the roads with little safety and security measures in place. The aim of this study was to determine the prevalence of high-risk behaviours, road crashes and the factors contributing to road crashes among commercial drivers.

**Methods:**

A cross-sectional study on commercial drivers was conducted at largest government bus/truck station of Peshawar, Pakistan. All these bus and truck stands are government registered vehicles stands. The duration of the study was six months, starting from October 2015 to March 2016. Multistage sampling technique was used as a sampling technique. Information was collected from 400 commercial drivers through a structured questionnaire about socio-demographics, high-risk driving behaviours, driving hours, use of drugs while driving, vehicle maintenance and crash involvement. Univariate logistic regression was applied for the association and Multiple logistic regression analysis was carried out to determine the potential confounders association between accidents and independent variables like over speeding, poor vision, use of cell phone while driving, use of seat belts. STATA 13.1 was used for analysis.

**Results:**

Overall, 400 commercial drivers were included in the study with the mean age of 38±7.7years. The minimum age of the driver was 18 years and maximum age 56 years’ with41% drivers between age group 31 to 40 years. Almost half of the drivers (44%) had primary education and around half (54%) were unable to read traffic sign boards. Around one third (31%) of the commercial drivers had road traffic accidents in the past which were highest large busses (44%) followed by Toyota Hiace (34%). Regarding high-risk behaviour; around one fifth (19%) of the drivers reported overspending, two third (66%) used cell phone while driving, with (39.5%) drivers use cannabis while driving. Multiple logistic regression analysis showed that over speeding(OR= 3.8, 95% CI 2.58 — 5.64, P = 0.001), driving at night (OR = 2.2, 95% CI 1.19 — 4.06, P = 0.01) and poor vision(OR = 2.59, 95% CI 1.23—5.46, P = 0.012)were associated with RTAs while age (OR = 0.60, 95% CI 0.42—0.85, P = 0.004) and valid license(OR = 0.41, 95% CI 0.23—0.72, P =0.002) were protective.

**Conclusions:**

High-risk behaviours like over speeding, running red lights, lack of valid driving license and poor vision were found to be strongly associated with road traffic accidents. These high-risk behaviours are prevalent in commercial drivers of Pakistan. Strict check and balance from traffic police are needed to overcome these high-risk behaviours.

**Keywords:**

Road traffic accident, Commercial drivers, High-risk behaviours

